# Multiple Congenital Osteochondromata

**Published:** 1916-03

**Authors:** 


**Multiple Congenital Osteochondromata.** Carman and Fisher, Ann. of Surg., Feb. 1915, report a case in a white man aged 30. Increase in size had been noted since 22. Bence-Jones body (albumose?) absent from urine, repeatedly. X-rays apparently show cystic nature of all tumors.

